# The use of immersive virtual reality technology in dementia care education for nursing students: A scoping review protocol

**DOI:** 10.1371/journal.pone.0342783

**Published:** 2026-03-03

**Authors:** Joey Oi Yee Wong, Lillian Hung, Alison Phinney, Winnie Sun

**Affiliations:** 1 School of Nursing, The University of British Columbia, Vancouver, British Columbia, Canada; 2 Faculty of Health Sciences, Ontario Tech University, Oshawa, Ontario, Canada; University of Porto Faculty of Medicine: Universidade do Porto Faculdade de Medicina, PORTUGAL

## Abstract

**Objective:**

This scoping review aims to identify and map the current available evidence regarding the use of immersive virtual reality technology in dementia care education for nursing students.

**Introduction:**

With the growing population living with dementia, there is an urgent need to prepare nurses for quality dementia care through providing relevant dementia care education for nursing students. Virtual reality is an emerging education medium for nursing education and dementia care education for nursing students. However, little evidence focuses on mapping how immersive virtual reality technology has been used in dementia care education for nursing students. With the increasing development and use of this technology, it is crucial to explore current evidence regarding the content and learning outcomes, design and development, and implementation of immersive virtual reality technology in dementia care education for nursing students.

**Inclusion criteria:**

Studies are eligible if they describe the use of immersive virtual reality technology in dementia education. The dementia care education must be designed for or targeted at nursing students or healthcare professional students (including nursing students) within academic settings, such as nursing schools, universities, and colleges.

**Methods:**

The proposed scoping review will be conducted following the Joanna Briggs Institute methodology for scoping reviews and the Population, Concept and Context (PCC) framework. The review will search CINAHL, MEDLINE, PsycINFO, Scopus, Web of Science and ProQuest Dissertations and Theses Global. Grey literature search will be conducted via Google Scholar. Extracted data on authors, country, study design, setting, and findings relevant to the review objective will be presented in a data extraction table, accompanied by a quantitative summary of studies’ characteristics and narrative summary. The PRISMAS-ScR figure will detail the selection process.

**Review registration number:**

This study has been registered with the Open Science Forum (OSF).

## Introduction

Over 57 million people were living with dementia in 2021 [1]. The global prevalence of people living with dementia is projected to increase significantly, reaching 139 million by 2050 [2]. To address the emerging and diverse needs of this population, the WHO’s global action plan on the public health response to dementia specifically emphasizes building the capacity of all health and social care professionals [3]. This priority ensures they receive “culturally appropriate, evidence-based and human rights-oriented” training through medical and paramedical pre-service and in-service programs [3].

The use of immersive virtual reality (VR) is emerging in nursing education, with VR equipment becoming more accessible and affordable to supplement traditional education. There are various definitions of VR. For this review, VR is defined as fully immersive VR that requires a head-mounted device. Immersive VR provides nursing students with a simulated, 3-dimensional, and safe environment to learn and experience, without worrying about unintentionally harming patients. Users will experience a sense of immersion, presence, and interactivity in the VR environment through the use of headsets or by being in a 360-audiovisual room [4]. A systematic review and a scoping review reported the learning outcomes of immersive VR use in nursing education, including improved empathy, learning anatomy and physiology, and enhanced clinical skills in intravenous catheter insertion, urinary catheterization, nasogastric tube feeding, and needlestick injury prevention [4,5]. While VR could provide authentic clinical experiences, the systematic review found several barriers to its use, including discomfort from wearing glasses with head-mounted devices, simulation sickness, and students’ unfamiliarity with VR technology [4]. The scoping review also suggested that VR is a new and growing area of nursing education, with a need to further explore how best to implement this technology and its strengths and limitations in nursing education [5]. The use of VR in dementia care nursing education was not covered in the two recent reviews.

There is a growing body of empirical studies on the use of immersive VR to teach nursing students about dementia care. For example, using immersive VR to develop empathy and attitudes among nursing students [6] and to embody the experience of a patient living with dementia through immersive VR [7]. With increasing interest in using immersive VR in dementia care education for nursing students, there is a lack of synthesis of (1) the content and learning outcomes, (2) design and development, and (3) implementation and pedagogical considerations of these education programs. There is a need for a review to systematically map these three domains of using immersive virtual reality technology in dementia care nursing education for students.

A preliminary search of MEDLINE, the Cochrane Database of Systematic Reviews, and JBI Evidence Synthesis was conducted on December 10, 2025. There were no current or ongoing scoping or systematic reviews on the topic of using immersive VR in dementia education for nursing students. Therefore, our scoping review aims to map the extent and nature of the current available evidence regarding the use of immersive virtual reality technology in dementia care education for nursing students.

### Review question

This scoping review will be guided by the following main review questions and sub-questions.

### Primary review question

What has been reported in the literature about the content and learning outcomes, design and development, and implementation of immersive virtual reality technology in dementia care nursing education for students?

### Secondary questions

1) What is the main content and intended learning outcomes of these immersive VR dementia education programs for nursing students?
2) How are these immersive virtual reality dementia care education programs designed, and which partners (e.g., academic faculty, technology developers, clinical experts, people with lived experiences) are involved in the development process and in what specific roles or capacities?
3) What pedagogical practices (e.g., structure and format of the education program, briefing and debriefing components), facilitators, barriers and considerations are reported for implementing these programs in academic settings?

## Methods

The scoping review methodology is chosen because it offers a systematic approach to identify the extent of the literature on a topic, examine how research is conducted in a particular field, and analyze knowledge gaps to inform future research [8]. The proposed scoping review will be conducted according to the JBI methodology for scoping reviews [8] and the Population, Concept and Context (PCC) framework [9]. The scoping review protocol has been registered on the Open Science Forum (OSF) (DOI: 10.17605/OSF.IO/Z3NHC).

### Inclusion criteria

#### Participants.

Studies will be included if they focus on dementia education designed for nursing students, including undergraduate nursing students and pre-registration or pre-licensed nurses, or for healthcare professional students in general, including nursing students.

#### Concept.

The core concept for this review is the use of immersive virtual reality technology. The immersive virtual reality in this review is defined as virtual reality that requires a head-mounted device and provides a sense of full immersion and presence.

#### Context.

Studies will be included if the focus is on dementia education, including any formal education or training relevant to diverse types of dementia, such as vascular dementia and frontotemporal dementia. The included studies about dementia education must also be designed for or targeted to be delivered in academic settings. Academic settings in this review refer to formal educational institutions, such as universities, colleges, and vocational schools, that provide dementia education for nursing students or healthcare professional students (including nursing students).

#### Types of evidence sources.

We will include studies of any design that addresses our research question. These study designs include both primary and secondary sources, e.g., qualitative, quantitative, and mixed methods studies. Systematic reviews and grey literature meeting the inclusion criteria will be included. There will be no restrictions based on the year of publication, the geographic location where the studies are conducted, or the type of publication (e.g., conference abstracts). To ensure accuracy in data extraction and analysis, this review will only include studies published in English and Chinese, as these are languages that the team members possess either native or professional proficiency. Non-English and non-Chinese studies will be excluded due to limited translation resources. This approach can minimize the risk of translation errors. There will be no limitations based on publication status. We will include both peer-reviewed evidence sources and grey literature. Refer to Table 1 for the inclusion and exclusion criteria.

**Table 1:** Inclusion and exclusion criteria.

InclusiTable 1. Inclusion and Exclusion Criteria.on criteria	Exclusion criteria
Population: Nursing students, pre-registration nurses, undergraduate nursing students, healthcare professional students (including nursing students)	Non-English and Non-Chinese studies
Concept: Use of immersive virtual reality technology, i.e., head-mounted devices as the education medium	
Context: The dementia care education must be designed for or targeted at nursing students or healthcare professional students (including nursing students) within academic settings, such as nursing schools, universities, and colleges	

### Search strategy

The search strategy will aim to identify both published and unpublished studies. We will use a three-step search strategy in this review. First, an initial search of MEDLINE (via EBSCOhost) and CINAHL (via EBSCOhost) was conducted to identify articles about the topic. A complete search strategy was then developed using the text words in the relevant articles’ titles and abstracts, and the index terms used to describe the articles. The comprehensive search strategy for the CINAHL (via EBSCOhost) database is shown in S1 File. The university librarian provided support for the initial search and for developing the full search strategy. The search strategy, including all identified keywords and index terms, will be tailored for each targeted database and information source. The reference list of the included sources, as well as relevant systematic and scoping reviews on similar topics related to dementia education, will be screened for additional studies. Searches will be conducted in December 2025.

### Information sources

The information sources include the following databases: CINAHL (via EBSCOhost), MEDLINE (via EBSCOhost), PsycINFO (via EBSCO), Scopus and Web of Science. ProQuest Dissertations and Theses Global will be used for a comprehensive search of academic theses and dissertations. Other relevant unpublished studies and grey literature, including relevant organizational reports and conference proceedings, will be searched using Google Scholar.

### Study/Source of evidence selection

All identified citations will be imported to Covidence following the search [9]. Covidence will be used for deduplication and screening. The duplicates will be screened by the reviewer JW manually and any non-duplicates identified will be returned to the pool for title and abstract screening. Any duplicates identified manually during the screening and extraction processes will be reported separately from duplicates identified by Covidence in the PRISMA flow diagram.

To ensure alignment, JW and LH will have a pre-screening calibration test on the same 50 titles and abstracts to reach a minimum of 80% inter-rater agreement [9] before the formal screening process begins. Following successful calibration, the titles and abstracts will be independently screened by the first author, JW, against the inclusion criteria for the review. Due to time and resource constraints, the second author, LH, will independently review a random 10–15% of the titles and abstracts separately to ensure and verify their accuracy [10]. Any conflicts among the dual-screened titles and abstracts will be resolved between JW and LH. If there are disagreements between JW and LH, AP/WS will be the third reviewer and make the final screening decision. The full text will be retrieved from potentially relevant sources.

The retrieved full text will be assessed against the inclusion criteria by JW. The literature will be excluded if it does not meet the inclusion criteria. To ensure alignment, JW and LH will perform a pre-screening calibration test on the same five full-text articles to achieve at least 80% inter-rater agreement [9] before JW begins the formal full-text screening. Due to time and resource constraints, LH will review a random 10–15% of the full text of selected citations separately [10]. Any disagreements between JW and LH arising from the dual-screened portion will be resolved through discussion. If the disagreement cannot be resolved, AP/WS will be the third reviewer and make the final screening decision.

### Data extraction

Data will be extracted with a data extraction table designed by the authors. The extraction table will include the following categories: first author and year of publication, country, type of literature, study design, setting, VR equipment used, content, design process of education programs, pedagogical practices, intended learning outcomes, impact, facilitators, barriers, and considerations for implementation in academic settings. See S2 File for the draft extraction table. JW and LH will independently conduct a pilot test of the data extraction table with two included studies. They will compare their extraction tables. Any disagreements between the two authors will be resolved through discussion or with the support of an additional author, AP/WS. The first author will then continue to extract the remaining data using the extraction table independently due to time and resource constraints. The data extraction table may be revised as necessary during the data extraction process. Any modifications that occur during the process will be documented in the final scoping review. We will identify and merge records reporting data from the same study, as identified during the screening and extraction process. We will identify the most recent record as the primary reference for reporting. Any relevant data from the related records will be reported under the primary reference in the data extraction table.

### Data analysis and presentation

The Preferred Reporting Items for Systematic Reviews and Meta-Analyses Extension for Scoping Reviews (PRISMA-ScR) flow diagram [11] will be used to detail the selection process of the scoping review and to present key information, including the sources and numbers of records identified, duplicated, screened, included, and excluded. The reasons for exclusion at full-text screening will be recorded. The number of excluded articles and reasons for exclusion will be documented. The extracted data and results of the identified existing literature will be systematically organized in the data extraction table in the final scoping review. Each column in the table will represent different key information, such as the year of publication, authors, study design, content, and intended learning outcomes. The articles will be organized chronologically in the table, from the latest to the oldest, to show the field’s evolution. The findings from the studies published in Chinese will be translated into English for reporting. The translation process will be done by two bilingual team members independently, with one member translating the findings from Chinese to English and another member back-translating from the English version back into Chinese. This process will allow the team to verify translation equivalence and accuracy for the final reporting and maintain the integrity of the review.

We will summarize the final records included through quantitative and descriptive synthesis. Quantitative synthesis will include descriptive statistics, such as frequency counts, to summarize study characteristics, including country and year of publication. Descriptive and narrative synthesis will be used to provide an overview of topics relevant to the review questions and to identify gaps in the evidence. According to JBI guidelines [10], the scoping review does not involve a critical appraisal of the included evidence. The absence of the critical quality appraisal does not imply endorsement of the methodological quality of the included evidence.

### Ethics and dissemination

The scoping review does not require research ethics approval or participants’ consent, as the data are publicly available. The findings will be disseminated through publication in an academic journal and presentations at conferences, allowing access for nursing and other healthcare educators, students from nursing and other healthcare disciplines, policymakers, people with lived experiences of dementia, and the public. The findings of the scoping review will inform and guide future research and practice in the field.

### Strengths and limitations

This scoping review will be rigorous, utilizing a search strategy developed in collaboration with an experienced medical librarian and covering a wide range of information sources and grey literature. However, certain limitations are anticipated. Due to time and resource constraints, the second reviewer will screen only a random 10–15% of titles, abstracts, and full texts, and will extract data from a small portion of the included studies. While this is an acceptable pragmatic approach for scoping reviews, it may introduce a potential risk of selection and extraction bias. To mitigate these risks, the team will conduct pre-screening calibration tests and pilot extractions to achieve inter-rater agreement before formal screening and data extraction. Furthermore, excluding non-English and non-Chinese studies due to limited translation resources may introduce language bias, particularly in the field of educational technology, where relevant studies may be published in other languages. This exclusion may limit the comprehensiveness of our evidence mapping.

## Supporting information

S1 File Search strategy for CINAHL (EBSCOhost). (DOCX)

S2 File Data extraction table. (DOCX)

S3 File Preferred reporting items for systematic review and meta-analysis protocols (PRISMA-P) 2015 Checklist. (DOCX)
