# Supplementary material for: The use of immersive virtual reality technology in dementia care education for nursing students: A scoping review protocol
Source: PLoS One. 2026 Mar 3;21(3):e0342783. doi: 10.1371/journal.pone.0342783 (PMC12956109; doi:10.1371/journal.pone.0342783)
Supplement: S1 File — (DOCX) [file pone.0342783.s001.docx]

### **S1 Appendix.**

### **Search strategy CINAHL (via EBSCOhost).**

| **Set** | **Search Terms** | **Search Modes** | **Results** |
| --- | --- | --- | --- |
| S1 | (MH "Dementia+") | Proximity | 91,690 |
| S2 | XB (alzheimer* OR "Cognitive disorder*" OR Dementia OR "Frontotemporal lobar degeneration" OR "Lewy body disease" OR "Major neurocognitive disorder*" OR "Memory loss*" OR "Mild cognitive impairment") | Proximity | 108,152 |
| S3 | S1 OR S2 | Proximity | 129,550 |
| S4 | (MH "Education+") | Proximity | 1,153,187 |
| S5 | (MH "Learning+") | Proximity | 152,595 |
| S6 | XB (Academia OR curricul* OR educat* OR learn* OR train*) | Proximity | 888,848 |
| S7 | S4 OR S5 OR S6 | Proximity | 1,702,319 |
| S8 | (MH "Virtual Reality+") | Proximity | 9,184 |
| S9 | XB "virtual realit*" OR XB VR | Proximity | 10,056 |
| S10 | XB "immersive technolog*" | Proximity | 109 |
| S11 | XB "virtual environment*" | Proximity | 1,598 |
| S12 | S8 OR S9 OR S10 OR S11 | Proximity | 15,486 |
| S13 | XB (health* care student* OR health profession* student* OR health* care profession* student*) | Proximity | 6,068 |
| S14 | (MH "Students, Nursing+") OR (MH "Students, Nursing, Practical") | Proximity | 53,233 |
| S15 | XB ("nurs* undergraduate*" OR "pre-register* nurse*") | Proximity | 944 |
| S16 | S13 OR S14 OR S15 | Proximity | 58,321 |
| S17 | S3 AND S7 AND S12 AND S16 | Proximity | 12 |

### Search conducted on December 10, 2025
