# Supplementary material for: The use of immersive virtual reality technology in dementia care education for nursing students: A scoping review protocol
Source: PLoS One. 2026 Mar 3;21(3):e0342783. doi: 10.1371/journal.pone.0342783 (PMC12956109; doi:10.1371/journal.pone.0342783)
Supplement: S2 File — (DOCX) [file pone.0342783.s002.docx]

### **S2 Appendix.**

### **Data extraction instrument.**

| Article (Author, year, country) | Type of literature | Study Design, Setting and Participants | VR equipment used | | Content | Intended learning outcomes | | Design process of the education program | | Pedagogical practices | Impact | Facilitators, barriers and considerations for implementation in academic settings |
| --- | --- | --- | --- | --- | --- | --- | --- | --- | --- | --- | --- | --- |
| Author, year, country |  |  |  |  | | |  | |  |  |  |  |
| Author, year, country  Author, year, country  Author, year, country  Author, year, country  …... |  |  |  |  | | |  | |  |  |  |  |
